# Inference of Crosstalk Effects between DNA Methylation and lncRNA Regulation in NSCLC

**DOI:** 10.1155/2018/7602794

**Published:** 2018-06-24

**Authors:** Binhua Tang

**Affiliations:** ^1^Epigenetics & Function Group, School of the Internet of Things, Hohai University, Jiangsu 213022, China; ^2^School of Public Health, Shanghai Jiao Tong University, Shanghai 200025, China

## Abstract

Intercellular crosstalk effects between DNA methylation and lncRNA regulation remain elusive in lung carcinoma epigenetics. We present an application toolkit MetLnc in integration and annotation for group-wise NSCLC tissue-based DNA methylation and lncRNA profiling resources, to comprehensively analyze differentially methylated loci and lncRNAs through genome-wide analysis. Together with multiple analytic functions, MetLnc acts as an efficient approach on epigenetic omics integration and interrogation. Via the benchmark with group-wise NSCLC tissue profiling and TCGA cohort resources, we study differentially methylated CpG loci and lncRNAs as meaningful clues for inferring crosstalk effects between DNA methylation and lncRNA regulation; together we conclude with investigated biomarkers for further epigenetics and clinical trial research.

## 1. Introduction

Comprehensive interrogation and genome-wide annotation of intercellular differential DNA methylation and its association in long noncoding RNAs (lncRNA) regulation are open questions in carcinoma epigenetics studies [1–3].

Genome-wide DNA methylation analysis across multiple samples, especially the recent pan-cancer study can retrieve cell- and tissue-specific characteristics with identification of differentially methylated loci and regions [4–7].

Increasing evidences highlight lncRNA as crucial regulators with essential cancer cell functions in cell proliferation, apoptosis, and metastasis. LncRNAs can regulate transcription and posttranscription through diverse biological mechanisms in carcinogenesis [8–11].

Till now, several tools and databases were proposed concerning functional curation and analysis of lncRNA roles in cancer and disease, for example, NONCODE [12], Lnc2Cancer [13], and LncRNADisease [14], but there still lacks systematic investigation of lncRNA regulatory activities under other epigenetic contexts, such as DNA methylation, in development and progression of lung carcinoma.

Currently, NSCLC (non-small-cell lung carcinoma) accounts for more than 85% lung carcinoma occurrences, and NSCLC is relatively insensitive to clinical chemotherapy, compared to small cell carcinoma [15–17]. Herein based on 24 group-wise (paired) NSCLC patient tissue samples (12 carcinoma versus 12 paracarcinoma), we integrate the corresponding lncRNA and DNA methylation profiling information to study the underlying regulatory mechanisms by our developed toolkit, MetLnc.

MetLnc is designed for integrating lncRNA and HumanMethylation 450K Beadchip assay information [18–20], together with the suitable format from such platform as the Reduced Representation Bisulfite Sequencing (RRBS) profiling [7, 21–23]. MetLnc can implement intercellular interrogation of lncRNA and DNA methylation status among multiple samples, perform statistical analysis on methylated CpG loci and regions, and yield integrative visualization for the analysis results.

Together our developed toolkit performs benchmark analyses with the group-wise NSCLC patient tissue profiling and TCGA cohort resources, thus it is proved to be a versatile analytic approach in carcinoma epigenetics study.

## 2. Materials and Methods

### 2.1. Structure and Function Composed in MetLnc

**Figure 1:**
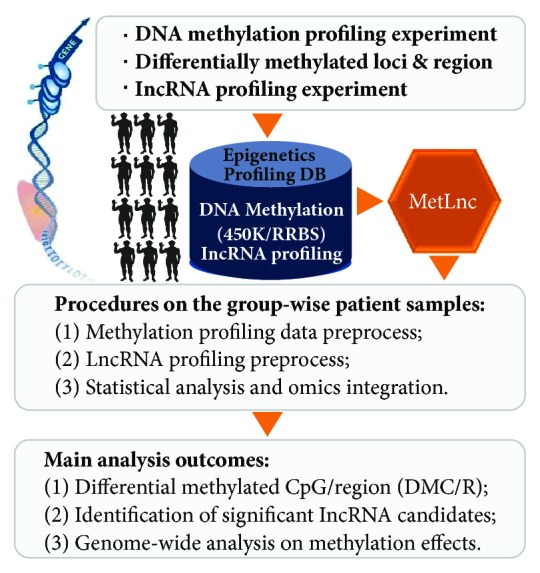
Schematic illustration for MetLnc process and outcomes. The toolkit carries out three main functional processes, namely, DNA methylation profiling preprocess (group-wise patient sample curation and profiling data formatting), information retrieval (CpG annotation, differential methylated CpG loci, and differential lncRNA candidates), and integration (genome-wide comparison and association analysis on the methylation effects).

The function and analysis flow in MetLnc are depicted in Figure 1, which covers main steps in processing DNA methylation and lncRNA profiling information, differentially expressed loci annotation and multi-source omics integration.

MetLnc is designed to carry out three main functional processes, namely, (I) DNA methylation and lncRNA profiling preprocess, including group-wise patient sample curation and profiling data format; (II) epigenetic information retrieval, including CpG annotation, differentially methylated CpG loci, and differentially expressed lncRNA candidates; and (III) knowledge integration and discovery, genome-wide comparison, and association analysis on the methylation and lncRNA effects on regulation. And we deposited the toolkit on GitHub (github.com/gladex/MetLnc/) under GNU General Public License v3.0.

### 2.2. NSCLC Sample Source for Profiling Experiments

The profiling experiments were carried out on the group-wise 12 tumor and 12 paratumor NSCLC tissues, by the collaborated lab in a NSCLC pilot project.

After the preprocessing and purification on the sample cells, DNA methylation and lncRNA experiments further profiled the corresponding methylation level and lncRNA expression level to investigate the crosstalk effects between the two epigenetic factors.

DNA methylation status profiling was implemented using Illumina Infinium HumanMethylation450 BeadChip platform for 24 paired NSCLC patient samples (carcinoma versus paracarcinoma tissues). lncRNA profiling was carried out based on Illumina HiSeq-2000 RNA Sequencing platform.

### 2.3. Analysis of RNA Sequencing-Based lncRNA Profiling

The corresponding RNA-seq profiling analysis was implemented to identify totally 124,060 differentially expressed non-coding RNA (ncRNA) candidates, with the log2 fold change range [-8.551009, 7.349755] and the ncRNA length range from 10 bp to 1,699,000 bp, respectively. We further determined 1,468 significant differentially expressed lncRNAs with the length ≥ 200 bp, the log2 fold change ≥ 2, and corresponding adjusted p-value ≤ 0.05. Then the lncRNA targets were annotated with UCSC gene information for further downstream analysis.

### 2.4. DNA Methylation Distribution for Paired NSCLC Patient Tissues

We detected DNA methylation level for the group-wise NSCLC patient tissues utilizing Illumina Infinium HumanMethylation 450 BeadChip technique. Totally 485,577 probes were identified from the pairwise carcinoma (12 patient tissues) and paracarcinoma (12 patient tissues) group. After necessary preprocess, total 6,163 probes were determined as significant hyper-/hypo- differentially methylated CpG sites (SDMC), with the absolute methylation difference percentage ≥ 0.25 and corresponding adjusted p-value ≤ 0.05. And we further annotate those 6,163 significantly differentially methylated CpGs with UCSC genome information (including SNP, promoter, and enhancer annotation), together with CpG island (shore) information. Thus, we found there are 723 out of 6163 (11.73%) SDMCs overlap with the known SNP annotation, 4,947 (80.27%) SDMCs are identified from CpG island/shores regions, 1,693 (27.47%) SDMCs locate at enhancer regions, and 1,230 (19.96%) SDMCs are detected from DHS.

### 2.5. Integration of DNA Methylation and lncRNA Profiling Information

After the preprocessing DNA methylation and lncRNA profiling data, we further integrated both information based on the genomic location, namely, considering differentially methylated loci within promoter region (TSS±1000 bp) and body region of differentially expressed lncRNA candidates.

Together we further identified the potential targeted genes based on the cis-regulation of lncRNAs; namely, we only consider targeted genes where their promoter region (TSS±1000 bp) has differentially expressed lncRNAs.

## 3. Comprehensive Analysis and Functional Annotation

### 3.1. Differential Analysis for Paired NSCLC Patient Tissues

We further studied the crosstalk mechanism of DNA methylation and lncRNA on transcription regulation. We performed the profiling experiments on the group-wise 12 tumor and 12 paratumor NSCLC tissues.

**Figure 2:**
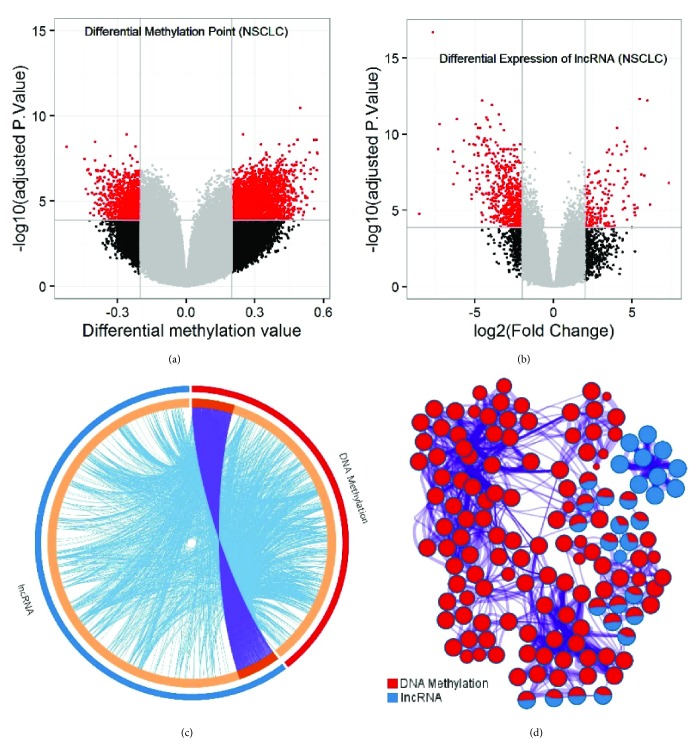
Meta-analysis on the lncRNA and DNA methylation profiling experiments in the group-wise NSCLC samples (tumor and paratumor NSCLC tissues). (a) and (b) Volcano plots for DNA methylation and lncRNA profiling on the group-wise NSCLC tissue samples. (c) Circos diagram indicating the overlap status between DNA methylation and lncRNA regulated genes, where blue curves denote the GO association among the gene candidates, and purple curves for the overlap level in gene count; (d) the ontology enrichment network between DNA methylation and lncRNA regulated genes.

After the sample normalization and necessary data preprocessing, we investigated their differential expression status of lncRNA and DNA methylation and performed meta-analysis on the identified candidates with coexpression activities comprehensively; see Figure 2.

Differential expression and annotation analysis on both DNA methylation and lncRNA identified that 6,163 significantly differentially methylated CpGs have 1,339 targeted genes, and 1,468 significant differentially expressed lncRNAs have 2,035 targeted genes, Figures 2(a) and 2(b).

Within the two targeted gene groups, there are 164 common targeted genes. Inside the Circos diagram, Figure 2(c), each arc represents a gene list, where each gene has a spot on the arc. Dark orange color represents the genes that appear in multiple lists and light orange color represents genes that are unique to that gene list. Purple lines link the same genes shared by multiple gene lists.

Through the Gene Ontology analysis on the differentially expressed candidates, we found that DNA methylation has the overwhelming effects on the entire regulation process, compared with lncRNA, although DNA methylation has the relatively lower targeted genes (1,339) than lncRNA (2,035), Figure 2(d); and the ontology enrichment network with its nodes displayed as pies, and the pie sector is proportional to the number of hits originated from a gene list related to DNA methylation (red) or lncRNA (blue), respectively.

We further interrogated the ontology distribution for the 164 common candidate genes regulated by DNA methylation and lncRNA and found their ontology terms cover the below processes that can be clustered into four major groups, namely, (I) negative regulation of development (multicellular organismal process/cell differentiation); (II) epithelial cell development (differentiation); (III) (trans-)synaptic signaling/synaptic transmission; (IV) muscle organ/tissue development.

The above phenomena indicate that DNA methylation and lncRNA in NSCLC mainly initiate the crosstalk activities in development and differentiation stages at epithelial cell and synaptic levels. We wonder whether such clues have any linkage with clinical outcomes in lung carcinoma.

### 3.2. Clinical Outcome by the Crosstalk between DNA Methylation and lncRNA

Thus, we retrieved the corresponding LUAD (Lung Adenocarcinoma, with 506 patient cohort) and LUSC (Lung Squamous Cell Carcinoma, with 495 patient cohort) clinical information from TCGA (The Cancer Genome Atlas) resources [5, 17] and further interrogated the clinical outcome association for the differentially expressed candidate genes, which are putatively regulated by DNA methylation and lncRNA.

Furthermore, to avoid the heterogeneity problem within tissue samples, we utilized z-score method to normalize the TCGA expression data and selected z-score = ±1.96 as the differential expression threshold; thus, on each gene candidate, we can further associate its differential expression status with clinical outcome by means of Kaplan-Meier survival analysis approach [24, 25].

For simplicity, we selected four representative candidate genes, namely, NTM (11q25, Entrez:50863), SIGLEC12 (19q13.41, Entrez:89858), TNXB (6p21.32, Entrez:7148), and LIMCH1 (4p13, Entrez:22998) as study cases.

**Figure 3:**
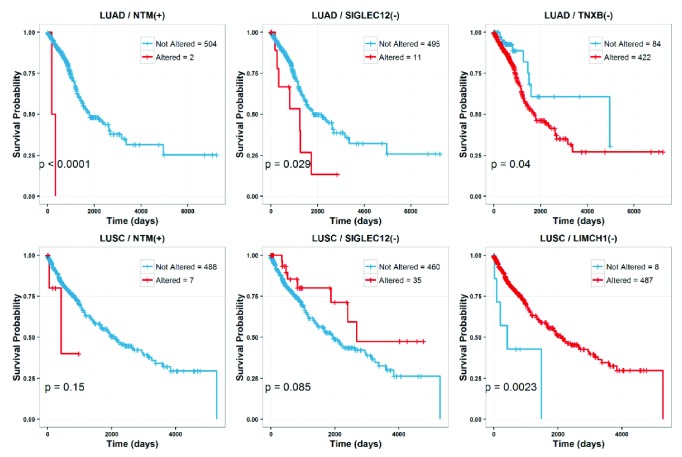
Clinical outcome analysis on the meta-analysis on the lncRNA and DNA methylation profiling experiments in the group-wise NSCLC tissue samples (tumor and paratumor NSCLC tissues).

Figure 3 illustrates the analysis results for LUAD (top) and LUSC (bottom) cases with calculated log-rank test p-values at the bottom left panel, respectively.

Comparatively, we found that NTM can act as clinical signature for both LUAD and LUSC, the entry of 488 patients with not altered status has better clinical outcomes than the altered entry; SIGLEC12 also works as signature for LUAD and LUSC, but the entry in LUAD with altered status has worse clinical outcome than that in LUSC; similarly, for TNXB, the entry in LUAD with altered status has worse clinical outcome; and LIMCH1 in LUSC indicates a better outcome for 487 patients with altered status.

Generally, the analyses reveal that the four gene candidates can work as potential lung cancer biomarkers, and TNXB and LIMCH1 are specifically to LUAD and LUSC, respectively.

## 4. Discussion and Conclusion

Intercellular crosstalk effects between DNA methylation and lncRNA regulation remain open questions in carcinoma epigenetics, although both epigenetic regulations are commonly regarded as important factors in cell differentiation and development processes.

We present an application toolkit MetLnc in integration and annotation for DNA methylation and lncRNA, thus to comprehensively analyze differentially methylated loci (regions) and lncRNA transcription through deep interrogation. MetLnc provides multiple versatile functions for investigating and annotating DNA methylation profiles. Generally, the toolkit can act as a comprehensive approach on epigenetic omics integration and deep interrogation.

Furthermore, through benchmark with NSCLC tissue profiling resources, we interrogate differentially methylated CpG loci and lncRNA as clues for inferring crosstalk effect between DNA methylation and lncRNA regulation.

Together via the clinical association analysis with TCGA cohort resources, we further determined the clinical outcome association for the identified differentially expressed genes, which are putatively regulated by DNA methylation and lncRNA. We found the identified gene candidates can work as potential lung cancer biomarkers; especially TNXB and LIMCH1 have clinical survival specificity to LUAD and LUSC, respectively. Such discovery has meaningful implications to cancer epigenetics and further clinical trial studies.

## Data Availability

The data used to support the findings of this study are available from the corresponding author upon request.

## Abbreviations

LncRNA: Long non-coding RNA
NSCLC: Non-small-cell lung carcinoma
LUAD: Lung adenocarcinoma
LUSC: Lung squamous cell carcinoma
TCGA: The Cancer Genome Atlas.
